# Long-term treatment of an ischemic jejunal stricture: Is stenting a viable option?

**DOI:** 10.1016/j.vgie.2022.05.005

**Published:** 2022-08-16

**Authors:** Andrew Canakis, Shayan S. Irani

**Affiliations:** 1 Division of Gastroenterology and Hepatology, University of Maryland School of Medicine, Baltimore, Maryland; 2 Division of Gastroenterology and Hepatology, Virginia Mason Medical Center, Seattle, Washington

**Keywords:** BMI, body mass index, FCSEMS, fully covered self-expanding metal stent, PEG, percutaneous endoscopic gastrostomy, TPN, total parenteral nutrition

## Abstract

Video 1 Management of jejunal stricture with stenting.

Management of jejunal stricture with stenting.

## Introduction

Small-bowel strictures can present with variable patterns of obstructive symptoms. Determining the etiology can guide the appropriate management. Anastomotic or postsurgical causes from open abdominal surgeries can increase the risk of occurrence. In the setting of long, complex strictures, surgery is the mainstay of treatment.^1^ In addition to surgically related adverse events, more than 70% of patients can develop recurrent strictures significantly increasing the risk of malnutrition and short bowel syndrome.^2^^,^^3^ Endoscopic management with balloon dilation has been used in uncomplicated short length (<5 cm) strictures, although there is a risk of perforation.^1^^,^^3^

The fibrostenotic changes around a stricture may be amenable to stenting. Although no fully covered self-expanding metal stents (FCSEMSs) are available for enteral stenting in the United States, biliary and esophageal stents could be repurposed.^4^, ^5^, ^6^, ^7^ However, their migration rates can be very high; securing them to a percutaneous endoscopic gastrostomy (PEG) tube with a suture can reduce their inward migration.^8^ We present a case in which we managed a long, ulcerated, ischemic jejunal stricture with an FCSEMS (Video 1, available online at www.giejournal.org).

## Case

**Figure 1:**
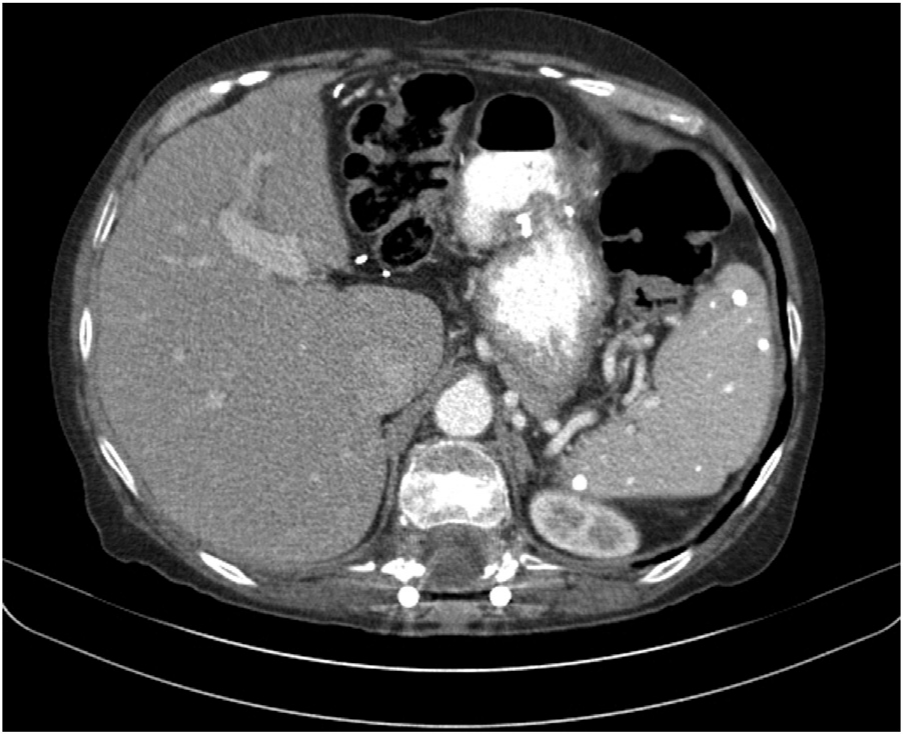
Abdominal CT scan showing a thickened elongated jejunal stricture past the gastrojejunostomy.

**Figure 2:**
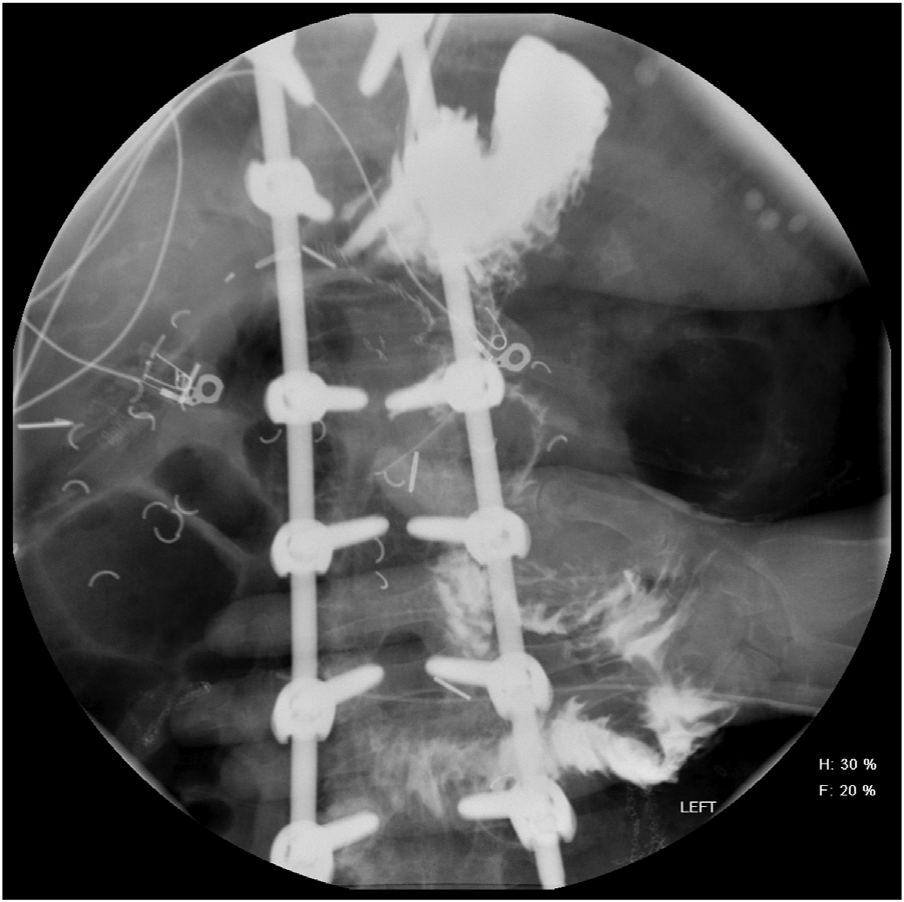
Upper GI stricture showing a thickened elongated jejunal stricture past the gastrojejunostomy.

A 71-year-old woman with a history notable for 7 prior surgeries, including a biliopancreatic diversion, left and right colectomy for colon cancer, and laparotomy with small-bowel resection, was referred for nausea, vomiting, abdominal pain, and weight loss. Her symptom onset was after her last surgery 9 months prior, progressing over the last 2 months with inability to tolerate per os intake, a body mass index (BMI) of 17.5, and an albumin of 2.2 g/dL requiring total parenteral nutrition (TPN) to nourish her. Her BMI prior to her first weight loss surgery was 41.6. An abdominal CT scan and upper GI series showed a thickened elongated jejunal stricture, just beyond the gastrojejunostomy, suspicious for an ischemic etiology (no nonsteroidal anti-inflammatory drug use or inflammatory bowel disease) (Figs. 1 and 2).

She was not deemed an ideal operative candidate by 2 bariatric surgeons and was referred for endoscopic management.

**Figure 3:**
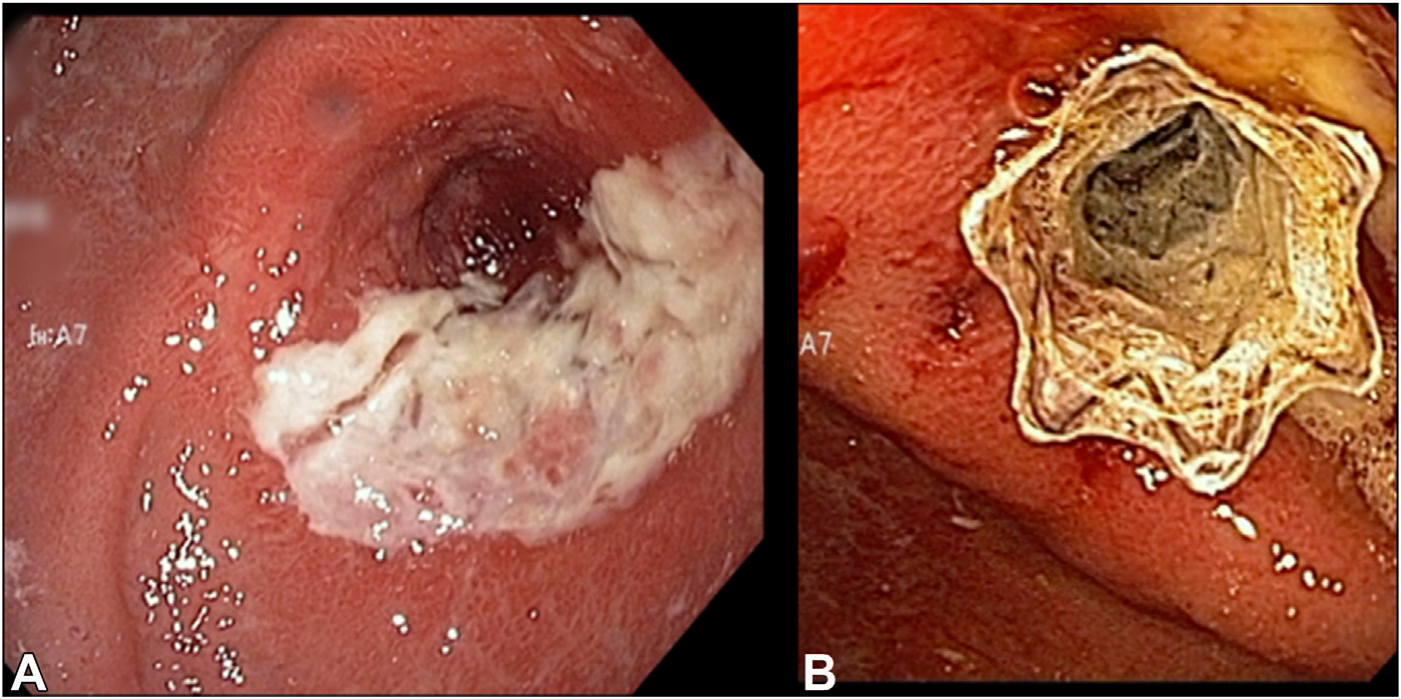
Endoscopic view of the initial stricture before **(A)** and after **(B)** stent placement.

**Figure 4:**
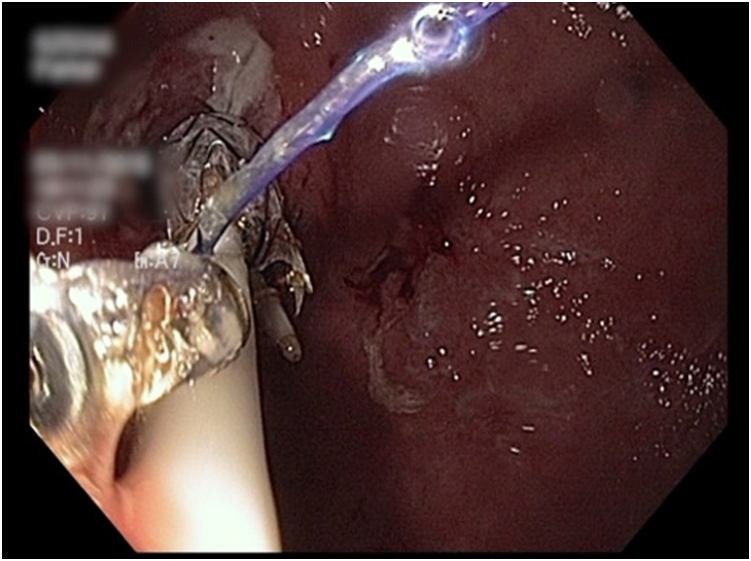
The stent was sutured using a single suture and secured to a percutaneous endoscopic gastrostomy tube to prevent inward migration.

**Figure 5:**
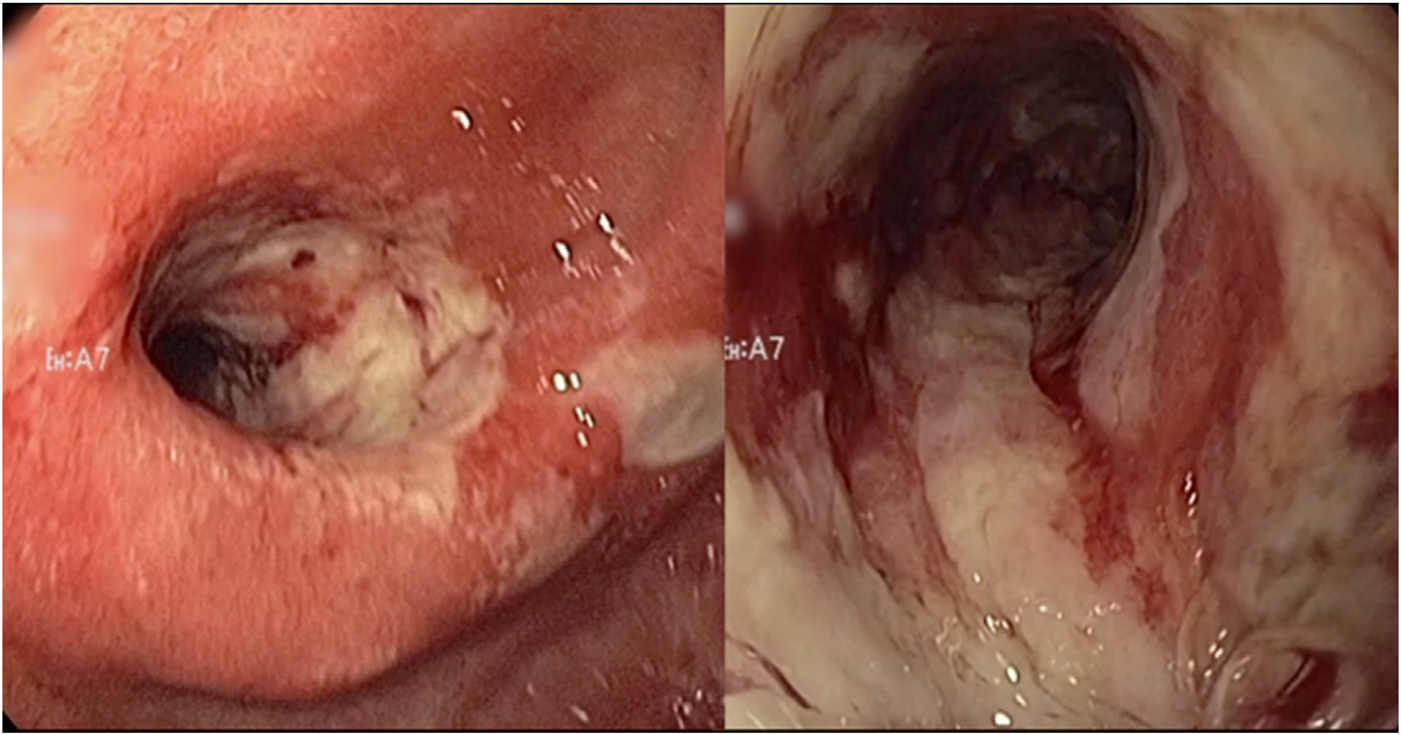
Stricture after initial stent removal.

**Figure 6:**
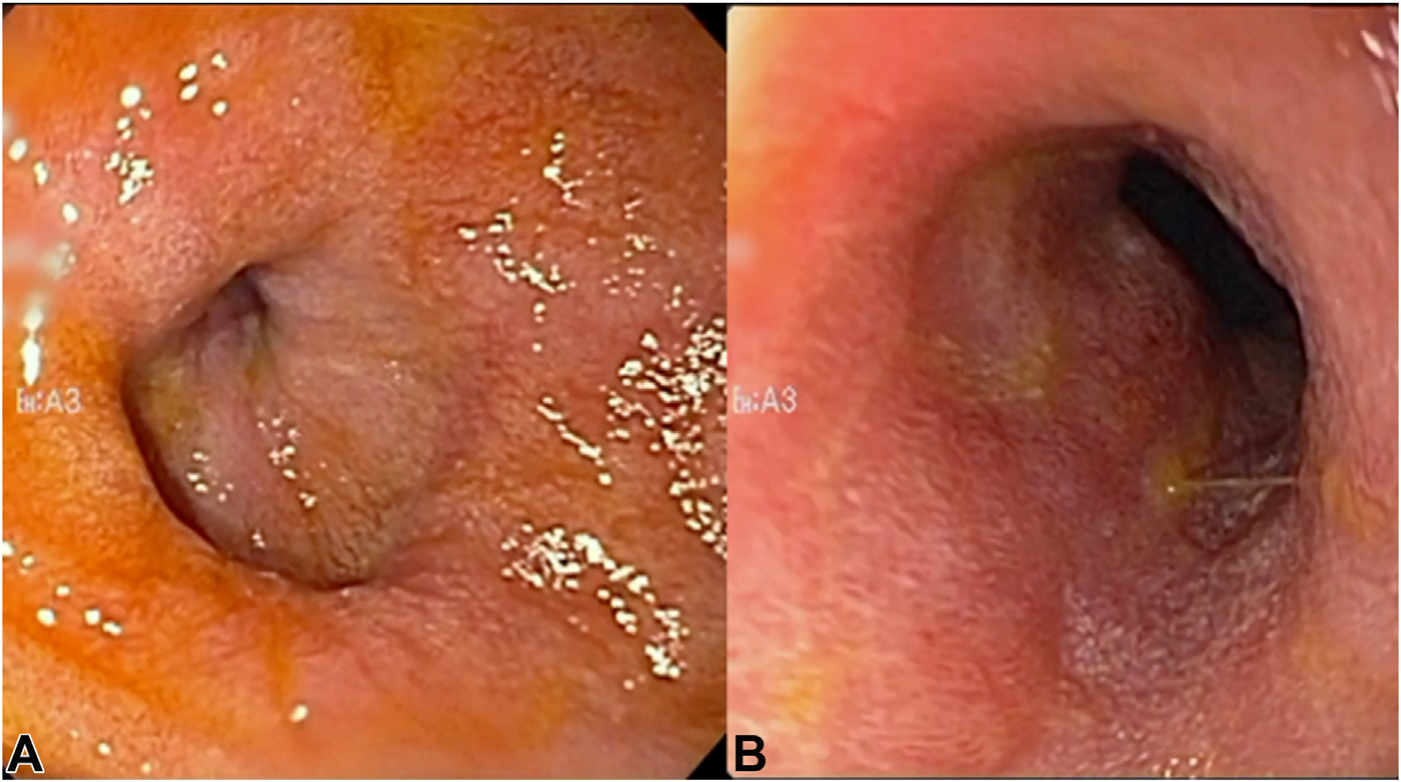
Repeat endoscopy 5 years later before **(A)** and after **(B)** balloon dilation to 15 mm.

During the endoscopy, a 5-cm-long (3-mm internal diameter) deeply ulcerated stricture, also consistent with an ischemic stricture, was identified. Balloon dilation seemed high risk, and thus a 10- × 10-cm biliary FCSEMS (Gore, Viabil, Ariz, USA) was placed (Fig. 3). The stent was then sutured using a single suture and secured to a PEG tube to prevent inward migration (Fig. 4). Jejunal feeds were started, TPN was stopped, and she was discharged on a full liquid diet. The stent was upsized to 14 × 10 cm (Alimaxx-B, CMeritt-Endotek, South Jordan, Utah) (Fig. 5) 41 days later, She was transitioned to a low-residue diet and successfully weaned off jejunal feeds within 4 weeks. Three months later, the stent was upsized to 16 × 10 cm and then 18 × 6 cm (Niti-S, Taewoong, Seoul, Korea). The 18-cm self-expanding metal stent migrated into the stomach and was removed 52 days later uneventfully with stricture resolution. She remained asymptomatic for 2 years until she developed mild symptoms of nausea and vomiting. A mild recurrence of the stricture, but with complete mucosal healing, was noted, and was easily dilated using a 15-mm balloon (Fig. 6). Throughout a 6-year follow-up, she has consumed a mostly unrestricted diet, needed only 1 additional dilation, and has maintained a BMI of 29.5 without significant symptoms.

## Conclusion

Repurposing biliary and esophageal FCSEMSs and securing them to a PEG can allow treatment and healing of a complex ischemic stricture with a good long-term clinical outcome. Further studies are needed to support the widespread applicability of this approach.

## Disclosure

*Dr Irani is a consultant for Boston Scientific and Gore. Dr Canakis declares no relevant funding for this work.*

## Supplementary data

Video 1 Management of jejunal stricture with stenting.
